# Predicting Gut Microbiome Diversity and Core Species From the Urine Metabolome

**DOI:** 10.1002/mco2.70950

**Published:** 2026-08-19

**Authors:** Yu Lin, Jiaqi Zhu, Alessia Visconti, Kari Wong, Gregory Michelotti, Tim D. Spector, Mario Falchi, Ana M. Valdes, Panayiotis Louca, Cristina Menni

**Affiliations:** ^1^ Department of Twin Research Ageing and Genomic Epidemiology King's College London London UK; ^2^ Centre For Biostatistics, Epidemiology, and Public Health Department of Clinical and Biological Sciences University of Turin Turin Italy; ^3^ Metabolon Research Triangle Park Morrisville North Carolina USA; ^4^ Academic Rheumatology Clinical Sciences Building Nottingham City Hospital University of Nottingham Nottingham UK; ^5^ Department of Pathophysiology and Transplantation Università Degli Studi Di Milano Milan Italy; ^6^ Fondazione IRCCS Cà Granda Ospedale Maggiore Policlinico Angelo Bianchi Bonomi Hemophilia and Thrombosis Center Milan Italy

1

Dear Editor,

Urine is a commonly used biofluid for medical diagnostics, as it reflects a wide range of metabolic processes. Several urinary metabolites, including hippurate, formate, and 4‐hydroxyphenylacetate, have been found to correlate with gut microbiome diversity, and have been proposed as markers for metabolic health [1, 2].

Metabolites produced by the gut microbiota can cross the intestinal wall, pass through the liver and kidney, and consequently be detected in urine, thereby providing a potential window into gut microbial activity. Compared to plasma or serum, urine is a readily accessible non‐invasive biofluid with less interference from proteins and lipids.

In the human gut microbiome, alterations in gut microbial diversity and shifts in core bacterial taxa are closely associated with host health [2]. Therefore, urine metabolites may provide an indirect but informative way to predict gut microbiome alterations and identify gut dysbiosis in relation to host health, which is particularly relevant in settings where stool samples are unavailable. Although prior studies report associations between individual urinary metabolites and gut microbiome features [1, 2], evidence supporting urine metabolomics as a population‐level proxy for microbiome diversity and composition remains limited, particularly under rigorous out‐of‐sample validation. In this study, we aim to identify a urinary metabolic signature of gut microbiome diversity and composition.

This observational cross‐sectional study included 1195 women from the TwinsUK cohort, UK adult twin registry established to study ageing and common complex diseases, with concurrent measures of urinary metabolites (Metabolon inc) and gut microbiome composition (shotgun metagenomes). See Supporting Information Methods for details. Participants provided informed written consent, and the study was approved by the St. Thomas’ Hospital Research Ethics Committee (REC reference: EC04/015). Individuals were split 80/20 into training and testing sets using group‐based splitting and related individuals were assigned to the same set. In the training set, random forest models with five‐fold cross‐validation (5‐cv) were applied to classify participants into low versus high gut microbial diversity (bottom and top tertiles, selected to ensure balanced groups), measured by Shannon diversity, observed features, and Berger–Parker dominance. The model with optimal parameters was then evaluated in the testing set. Input features included metabolites with ≥ 80% prevalence (*n =* 782). We compared the predictive performance of models using metabolites alone, covariates alone (age and BMI), and metabolites combined with covariates.

To identify the most informative predictors of Shannon diversity, we ranked features by average Gini importance from the training‐set random forest models and applied forward selection to obtain a parsimonious metabolite panel that retained near‑maximal predictive accuracy. Associations between alpha diversity and the most predictive metabolites for Shannon diversity were assessed using linear mixed models adjusting for age, BMI, batch, and family structure in the full dataset.

To identify the core microbiome, we evaluated multiple detection and prevalence thresholds and selected the combination yielding a median number of core taxa. See Supporting Information Methods for details. We further applied random forest models to classify the top and bottom tertiles of each of the core taxon abundances, using both the full metabolite set and the 10 most predictive to prioritize capturing species‑specific metabolic signatures over strict model sparsity. See Supporting Information Methods for detailed statistical analysis.

We analyzed 1195 female participants from the TwinsUK (mean age: 58.96 ± 14.59 years; mean body mass index [BMI]: 25.95 ± 5.04 kg/m^2^). The gut microbiome had mean Shannon diversity of 3.84 ± 0.57, mean observed features of 297.51 ± 80.50, and mean Berger–Parker dominance score of 0.98 ± 0.0064. The models trained on combined features (metabolites, age, and BMI) and on metabolites alone had similar AUCs for Shannon (0.733 vs. 0.734) (Figure 1A), observed features (0.774 vs. 0.777), and Berger–Parker dominance (0.758 vs. 0.772). Importantly, the top six predictive metabolites for Shannon diversity achieved almost identical AUC compared to the full panel (AUC = 0.732), balancing a small number of metabolites with comparable performance. This metabolite panel also moderately differentiated low versus high observed features (AUC = 0.770) and Berger–Parker dominance (AUC = 0.752) (Figure 1A). All top six metabolites demonstrated robust associations with Shannon, observed features, and Berger–Parker dominance in the linear mixed models adjusting for age, BMI, batch, and family structure (Figure 1B).

**FIGURE 1:**
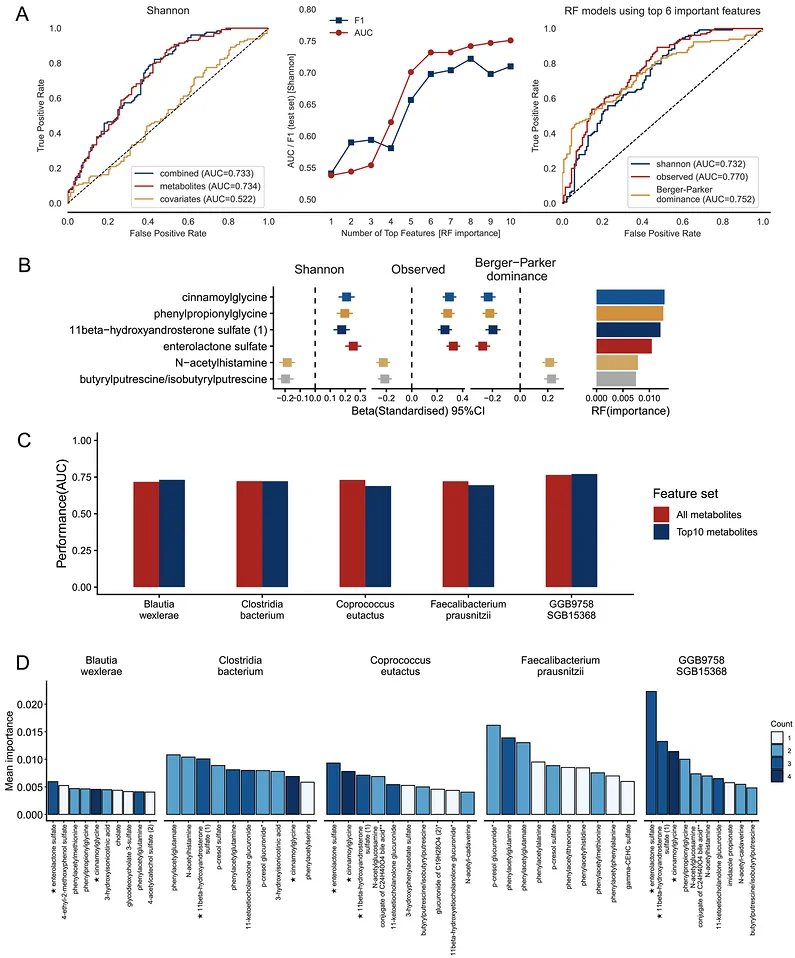
Prediction of alpha diversity using the urine metabolome. (A) Performance of prediction models for Shannon diversity. In the panel on the left, models were trained using different feature sets: covariates (age and body mass index [BMI]), urine metabolites, and a combination of covariates and metabolites. Receiver operating characteristic (ROC) curves were generated from the testing set. The middle panel shows prediction performance of random forest models with increasing number of urine metabolites. In the panel on the right, model performance for Shannon diversity, observed features, and dominance using the top six most important metabolites identified from the Shannon diversity model. RF: random forest, AUC: area under the ROC curve. (B) Associations between the top six metabolites and alpha diversity (Shannon diversity, observed features and Berger–Parker dominance). The three panels on the left show standardized beta coefficients estimated using linear mixed models, adjusting for age, BMI, batch and family structure. The panel on the right shows the average feature importance identified from random forest (RF) models using five‐fold cross‐validation. CI: confidence interval. (C) Prediction performance for five core microbial species. Comparison of the AUC for models trained using either all urinary metabolites or the top 10 metabolites selected from each species‐specific model. (D) The top ten metabolites contributing to the prediction for each species. Mean feature importance was derived from five‐fold cross‐validation in the training set. Bar colours indicate how frequently each metabolite appeared among the top ten features across models for different core microbial species. ★ indicates features shared with the most important predictors of Shannon diversity.

We next examined core bacterial species in the gut microbiome, identifying 44 core species with > 0.1% abundance and > 50% prevalence. Among 44 core taxa, five species achieved an average random forest AUC greater than 70% in the testing set: including *Blautia wexlerae* (71.8%), *Clostridia bacterium* (72.3%), *Coprococcus eutactus* (73.1%)*, Faecalibacterium prausnitzii* (72.2%), and *GGB9758 SGB15368* (76.5%) (Figure 1C). Models restricted to the top 10 metabolites for each species performed comparably to models using the full metabolite set. Among the top six metabolites for Shannon, three metabolites, cinnamoylglycine, 11beta‐hydroxyandrosterone sulfate (1), and enterolactone sulfate, were also identified as predictive features for at least three bacterial species (Figure 1D). These findings indicate that microbiome‐related information in urine is concentrated within a small, biologically interpretable metabolite panel.

Here, we identify a set of urinary metabolites that are associated with gut microbiome diversity and composition, suggesting their potential as non‐invasive biomarkers of gut health and dysbiosis. As the most predictive metabolite for Shannon, cinnamoylglycine, a metabolite derived from cinnamic acid reflecting the microbial metabolism of dietary polyphenols, also ranked among the top 10 important metabolites for *Blautia wexlerae, Clostridia bacterium, Coprococcus eutactus*, and *GGB9758 SGB15368*. A previous study found that the gut microbiome explained the largest proportion of variance in serum cinnamoylglycine levels compared to all other measured metabolites [3]. Note that 11beta‐hydroxyandrosterone sulfate, the third important predictor, is a biomarker of adrenal androgen metabolism, which may be influenced by the gut microbial steroid hormone metabolism, and is known to be elevated in females with polycystic ovary syndrome [4]. Enterolactone sulfate is a microbially derived metabolite of dietary lignans that exhibits estrogenic properties and contributes to anti‐inflammatory activity [5]. Collectively, metabolites linked to dietary habits and hormone metabolism were identified as major predictors of gut microbial diversity and core bacterial abundance, highlighting host‐gut microbiome metabolic coupling. The predictive performance observed indicates that microbiome‐derived signals are detectable in urine but constrained by host metabolic processing. We note some study limitations. First, our study population included predominantly middle‐aged females, so results may not be generalizable to men. Second, although our machine learning models identified urinary metabolites associated with gut microbiome features, the cross‐sectional nature of our study precludes causal inference. Future studies should validate these results in sex‐balanced, age‐diverse independent cohorts, and use experimental models to investigate the underlying biological mechanisms and causal relationships.

In conclusion, we found urinary metabolites may reflect gut microbiome diversity and composition, with diet‐ and hormone‐related metabolites showing particularly strong predictive power. Urine metabolomics may offer a non‐invasive, scalable proxy for gut microbiome characterisation, providing a population‐level benchmark and proof‐of‐concept framework for future external validation, longitudinal replication, and mechanistic investigation.

## Author Contributions

**Yu Lin**: conceptualization, methodology, formal analysis, writing – original draft preparation. **Jiaqi Zhu**: methodology, validation, writing – review and editing. **Alessia Visconti**: data curation, software, writing – review and editing. **Kari Wong**: resources, writing – review and editing. **Gregory Michelotti**: resources, writing – review and editing. **Tim D. Spector**: funding acquisition, writing – review and editing. **Mario Falchi**: data curation, writing – review and editing. **Ana M. Valdes**: methodology, writing – review and editing. **Panayiotis Louca**: methodology, writing – review and editing. **Cristina Menni**: conceptualization, methodology, formal analysis, writing – original draft preparation, supervision. All authors have read and approved the final manuscript.

## Funding Information

This research was funded in whole, or in part, by the UKRI (MR/Y010175/1 and MR/T004142/1), the Chronic Disease Research Foundation (CDRF), and the Wellcome Trust (212904/Z/18/Z). For the purpose of open access, the authors have applied a CC BY public copyright to any author accepted manuscript version arising from this submission. The Department of Twin Research received support from grants from the Wellcome Trust (212904/Z/18/Z) and the MRF (MRC)/BHF (BHF) Ancestry and Biological Informative Markers for Stratification of Hypertension (AIM‐HY; MR/M016560/1), European Union, CDRF, Zoe Global Ltd., the NIHR Clinical Research Facility, and Biomedical Research Centre (based at Guy's and St Thomas’ NHS Foundation Trust in partnership with King's College London). The TwinsUK study was also supported by grant funding from Tinnitus UK. C.M. is funded by the CDRF and by the Italian Ministry of Education and Research (MUR): Dipartimenti di Eccellenza Program 2023‐2027 and by the Italian Ministry of Health—Bando Ricerca Corrente. P.L. is funded by the CDRF. A.M.V. is supported by the National Institute for Health and Care Research Nottingham Biomedical Research Centre. Support for this work was also provided by UKRI/MRC grants (MR/W026813/1 and MR/Y010175/1) awarded to A.M.V. and C.M.

## Ethics Statement

This study was approved by St. Thomas’ Hospital Research Ethics Committee (REC Ref: EC04/015). Written informed consent was obtained from all participants.

## Conflicts of Interest

T.D.S. is a co‐found of ZOE Ltd. A.M.V. is a consultant to ZOE Ltd. T.D.S. has received options from ZOE Ltd. K.W. and G.M. are employees of Metabolon, Inc., but have no relevant financial or non‐financial interests to disclose. All other authors declare no conflicts of interests.

## Supporting information




**Supporting File 1**: mco270950‐sup‐0001‐SuppMat.docx

## Data Availability

The data used in this study are held by the Department of Twin Research at King's College London. The raw metagenomic sequence data used in this study have been deposited in the European Bioinformatics Institute European Nucleotide Archive database (accession code: PRJEB98467). The raw mass spectrometry data relating to TwinsUK samples have been deposited to the TwinsUK BioResource data management team. The TwinsUK BioResource is managed by the Twin Research Executive Access committee (TREC) at King's College London, which provides governance of access to TwinsUK data and samples. The data can be released to bona fide researchers using our normal procedures overseen by the Wellcome Trust and its guidelines as part of our core funding (http://twinsuk.ac.uk/resources‐for‐researchers/access‐our‐data/).
