# Supplementary material for: Predicting Gut Microbiome Diversity and Core Species From the Urine Metabolome
Source: MedComm (2020). 2026 Aug 19;7(9):e70950. doi: 10.1002/mco2.70950 (PMC13490920; doi:10.1002/mco2.70950)
Supplement: Supplementary file 1 [file MCO2-7-e70950-s001.docx]

Supplementary Documents

**Predicting Gut Microbiome Diversity and Core Species from the Urine Metabolome**

Yu Lin^1^, Jiaqi Zhu^1^, Alessia Visconti^1,2^, Kari Wong^3^, Gregory Michelotti^3^, Tim D Spector^1^, Mario Falchi^1^, Ana M Valdes^4^, Panayiotis Louca^1^, Cristina Menni^1,5,6*^

1. Department of Twin Research, Ageing and Genomic Epidemiology, King’s College London, London, England, SE1 7EH, United Kingdom
2. Centre for Biostatistics, Epidemiology, and Public Health, Department of Clinical and Biological Sciences, University of Turin, Turin, Italy
3. Metabolon, Research Triangle Park, Morrisville, NC 27560, USA
4. Academic Rheumatology Clinical Sciences Building, Nottingham City Hospital, University of Nottingham, Nottingham, United Kingdom
5. Department of Pathophysiology and Transplantation, Università Degli Studi di Milano, Via Francesco Sforza, 35, 20122 Milan, Italy
6. Fondazione IRCCS Cà Granda Ospedale Maggiore Policlinico, Angelo Bianchi Bonomi Hemophilia and Thrombosis Center, 20122 Milan, Italy

^*^Correspondence to: [cristina.menni@kcl.ac.uk](mailto:cristina.menni@kcl.ac.uk)

**Materials and Methods:**

**1. Gut microbiome and metabolomic profiling**

In TwinsUK, urine samples were collected during clinic visits, while stool samples were typically collected at home within one week of the visit^1^. While this gap may introduce random noise, both metabolomic and microbiome profiles are relatively stable over such short intervals^2, 3^. Urine Metabolites were profiled by Metabolon inc using UHPLC-MS/MS^4^. Metabolites were inverse-normal transformed, and missing values were imputed using minimum run-day measures. Shotgun metagenomic sequencing was performed on stool samples as previously described^5^. MetaPhlAn4 was used for taxonomic profiling of microbial communities at the species-level genome bin resolution.

**2. Core species identification**

To identify the core microbiome, we evaluated multiple combinations of detection thresholds and prevalence criteria and selected the combination resulting in a median number of core taxa^6^. The core microbiome (a detection threshold of 0.1% relative abundance and a prevalence cutoff of >50% across all samples) likely reflects the most stable components, with importance to host and ecological stability.

**3. Statistical analysis**

Participants were randomly assigned to training (80%) and testing (20%) datasets using group-based splitting to ensure that related individuals were allocated to the same dataset. Random forest models with five-fold group-based cross-validation were trained in the training dataset to predict the top and bottom tertiles of microbial alpha diversity and core microbial species abundance from urinary metabolites. Hyperparameters were optimised by grid search on the training set using the following combinations: n_estimators: [100, 300, 500], max_depth: [None, 5, 10, 20], min_samples_split: [2, 5, 10], max_features: [sqrt, log2]. Model performance was evaluated in a held-out testing set using the area under the receiver operating characteristic curve (AUC). Metabolite importance was ranked according to the mean Gini importance across cross-validation folds, and forward feature selection was performed to identify a parsimonious metabolite panel for Shannon diversity prediction. Associations between urinary metabolites and alpha diversity indices were assessed using linear mixed models, adjusting for age, body mass index (BMI), batch, and family structure. Standardised regression coefficients are reported for all linear mixed models. Machine learning analyses and statistical modelling were performed in Python 3.13 and R 4.5.0.

**Reference**

1.	Verdi S, Abbasian G, Bowyer RCE, et al. TwinsUK: The UK Adult Twin Registry Update. *Twin Res Hum Genet*. 2019;22(6):523–9.

2.	Bermingham KM, Brennan L, Segurado R, et al. Genetic and Environmental Contributions to Variation in the Stable Urinary NMR Metabolome over Time: A Classic Twin Study. *J Proteome Res*. 2021;20(8):3992–4000.

3.	Mehta RS, Abu-Ali GS, Drew DA, et al. Stability of the human faecal microbiome in a cohort of adult men. *Nat Microbiol*. 2018;3(3):347–55.

4.	Attaye I, Beynon-Cobb B, Louca P, et al. Cross-sectional analyses of metabolites across biological samples mediating dietary acid load and chronic kidney disease. *iScience*. 2024;27(3):109132.

5.	Pope R, Visconti A, Zhang X, et al. Faecal metabolites as a readout of habitual diet capture dietary interactions with the gut microbiome. *Nat Commun*. 2025;16(1):10051.

6.	Neu AT, Allen EE, Roy K. Defining and quantifying the core microbiome: Challenges and prospects. *Proc Natl Acad Sci U S A*. 2021;118(51).
